# 3-[(3,5-Di-*tert*-butyl-2-hydroxy­benzyl­idene)methyl­eneamino]benzonitrile

**DOI:** 10.1107/S1600536809014809

**Published:** 2009-04-30

**Authors:** Yong-Feng Zhao, Jin-Ping Xiong, Yu Zuo

**Affiliations:** a College of Materials Science and Engineering, Beijing University of Chemical Technology, Beijing 100029, People’s Republic of China

## Abstract

The mol­ecule of the title compound, C_22_H_26_N_2_O, displays a *trans* configuration with respect to the C=N double bond. The dihedral angle between the planes of the two aromatic rings is 26.30 (15)°. There is a strong intra­molecular O—H⋯N hydrogen bond between the imine and hydroxyl groups.

## Related literature

For general background on Schiff base coordination complexes, see: Weber *et al.* (2007 ▶); Chen *et al.* (2008 ▶); May *et al.* (2004 ▶). For double-bond-length data, see: Elmah *et al.* (1999 ▶).
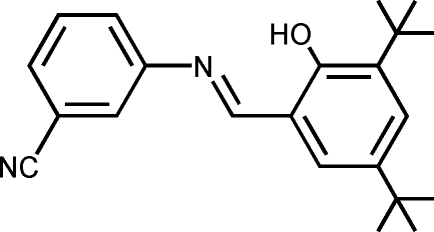


## Experimental

### 

#### Crystal data

C_22_H_26_N_2_O
*M* *_r_* = 334.45
Monoclinic, 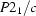

*a* = 14.897 (3) Å
*b* = 15.684 (3) Å
*c* = 8.8581 (18) Å
β = 97.86 (3)°
*V* = 2050.2 (7) Å^3^
*Z* = 4
Mo *K*α radiation
μ = 0.07 mm^−1^
*T* = 293 K
0.2 × 0.2 × 0.2 mm

#### Data collection

Rigaku Mercury2 diffractometer
Absorption correction: multi-scan (*CrystalClear*; Rigaku, 2005 ▶) *T*_min_ = 0.903, *T*_max_ = 1.000 (expected range = 0.891–0.987)
10436 measured reflections
3701 independent reflections
1746 reflections with *I* > 2σ(*I*)
*R*_int_ = 0.079

#### Refinement

*R*[*F* ^2^ > 2σ(*F* ^2^)] = 0.072
*wR*(*F* ^2^) = 0.189
*S* = 0.99
3701 reflections
230 parameters
H atoms treated by a mixture of independent and constrained refinement
Δρ_max_ = 0.14 e Å^−3^
Δρ_min_ = −0.15 e Å^−3^

### 

Data collection: *CrystalClear* (Rigaku, 2005 ▶); cell refinement: *CrystalClear*; data reduction: *CrystalClear*; program(s) used to solve structure: *SHELXS97* (Sheldrick, 2008 ▶); program(s) used to refine structure: *SHELXL97* (Sheldrick, 2008 ▶); molecular graphics: *SHELXTL* (Sheldrick, 2008 ▶); software used to prepare material for publication: *SHELXL97*.

## Supplementary Material

Crystal structure: contains datablocks I, global. DOI: 10.1107/S1600536809014809/gw2063sup1.cif

Structure factors: contains datablocks I. DOI: 10.1107/S1600536809014809/gw2063Isup2.hkl

Additional supplementary materials:  crystallographic information; 3D view; checkCIF report

## Figures and Tables

**Table 1:** Hydrogen-bond geometry (Å, °)

*D*—H⋯*A*	*D*—H	H⋯*A*	*D*⋯*A*	*D*—H⋯*A*
O1—H1*A*⋯N2	1.03 (5)	1.68 (5)	2.612 (3)	149 (4)

**Fig. 1.**
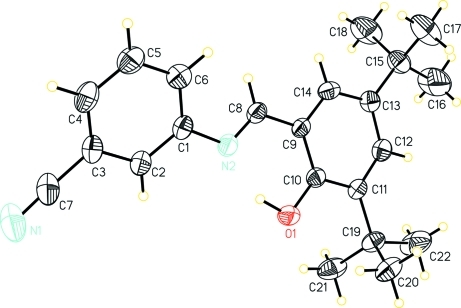
A view of the title compound with the atomic numbering scheme. Displacement ellipsoids are drawn at the 30% probability level.

## supplementary crystallographic information

### Comment

Schiff base compounds have received considerable attention for many years, primarily due to their importance in the development of coordination chemistry related to magnetism (Weber, *et al.*, 2007), catalysis (Chen, *et al.*, 2008) and biological process (May, *et al.*,2004). Our group is interested in the synthesis and preparation of Schiff base. Here, we report the synthesis and crystal structure of the title compound, (I).

Fig. 1 shows *ORTEP* plots of the title compounds. The dihedral angle between the mean planes of the two aromatic rings is 26.30 (0.15) ° showing that the Schiff-base ligand adopts a non-planar conformation. As expected, the molecule displays a *trans* configuration about the central C8=N2 function bond. The C8=N2 bond length of 1.286 (3)Å indicates a high degree of double-bond character comparable with the corresponding bond lengths in other Schiff bases (1.280 (2) Å; Elmah *et al.*, 1999). A strong intramolecular O–H···N hydrogen bond interaction is observed in the molecular structure.

### Experimental

All chemicals were obtained from commercial sources and used without further purification. 3-aminobenzonitrile (0.59 g, 5 mmol) and 3,5-di-*t*-butyl-2-hydroxybenzaldehyde (1.05 g, 4.5 mmol)were dissolved in ethanol (20 ml). The mixture was heated to reflux for 7 h, then cooled to room temperature the solution was filtered and after two weeks yellow crystals suitable for X-ray diffraction study were obtained. Yield: 1.27 g, 85%.

### Refinement

All the H atoms were found in the difference Fourier maps. The position of H1A is refined with the bond constraint O1—H1A = 0.82 Å.

### Crystal data

**Table:**

C_22_H_26_N_2_O	*F*(000) = 720
*M**_r_* = 334.45	*D*_x_ = 1.084 Mg m^−^^3^
Monoclinic, *P*2_1_/*c*	Mo *K*α radiation, λ = 0.71073 Å
Hall symbol: -P 2ybc	Cell parameters from 7104 reflections
*a* = 14.897 (3) Å	θ = 3.0–25.2°
*b* = 15.684 (3) Å	µ = 0.07 mm^−^^1^
*c* = 8.8581 (18) Å	*T* = 293 K
β = 97.86 (3)°	Prism, colorless
*V* = 2050.2 (7) Å^3^	0.2 × 0.2 × 0.2 mm
*Z* = 4	

### Data collection

**Table:**

Rigaku Mercury2 diffractometer	3701 independent reflections
Radiation source: fine-focus sealed tube	1746 reflections with *I* > 2σ(*I*)
graphite	*R*_int_ = 0.079
Detector resolution: 13.6612 pixels mm^-1^	θ_max_ = 25.2°, θ_min_ = 3.1°
ω scans	*h* = −17→17
Absorption correction: multi-scan (*CrystalClear*; Rigaku, 2005)	*k* = −14→18
*T*_min_ = 0.903, *T*_max_ = 1.000	*l* = −9→10
10436 measured reflections	

### Refinement

**Table:**

Refinement on *F*^2^	Primary atom site location: structure-invariant direct methods
Least-squares matrix: full	Secondary atom site location: difference Fourier map
*R*[*F*^2^ > 2σ(*F*^2^)] = 0.072	Hydrogen site location: inferred from neighbouring sites
*wR*(*F*^2^) = 0.189	H atoms treated by a mixture of independent and constrained refinement
*S* = 0.99	*w* = 1/[σ^2^(*F*_o_^2^) + (0.0819*P*)^2^] where *P* = (*F*_o_^2^ + 2*F*_c_^2^)/3
3701 reflections	(Δ/σ)_max_ = 0.004
230 parameters	Δρ_max_ = 0.14 e Å^−^^3^
0 restraints	Δρ_min_ = −0.15 e Å^−^^3^

### Special details

**Table:**

Geometry. All e.s.d.'s (except the e.s.d. in the dihedral angle between two l.s. planes) are estimated using the full covariance matrix. The cell e.s.d.'s are taken into account individually in the estimation of e.s.d.'s in distances, angles and torsion angles; correlations between e.s.d.'s in cell parameters are only used when they are defined by crystal symmetry. An approximate (isotropic) treatment of cell e.s.d.'s is used for estimating e.s.d.'s involving l.s. planes.
Refinement. Refinement of *F*^2^ against ALL reflections. The weighted *R*-factor *wR* and goodness of fit *S* are based on *F*^2^, conventional *R*-factors *R* are based on *F*, with *F* set to zero for negative *F*^2^. The threshold expression of *F*^2^ > σ(*F*^2^) is used only for calculating *R*-factors(gt) *etc*. and is not relevant to the choice of reflections for refinement. *R*-factors based on *F*^2^ are statistically about twice as large as those based on *F*, and *R*- factors based on ALL data will be even larger.

### Fractional atomic coordinates and isotropic or equivalent isotropic displacement parameters (Å^2^)

**Table:**

	*x*	*y*	*z*	*U*_iso_*/*U*_eq_	
O1	0.66011 (15)	0.15473 (13)	0.7833 (2)	0.0653 (6)	
C10	0.72011 (19)	0.08905 (18)	0.8159 (3)	0.0505 (8)	
N2	0.59185 (16)	0.11626 (15)	1.0309 (3)	0.0559 (7)	
C11	0.78194 (18)	0.06970 (18)	0.7144 (3)	0.0492 (8)	
C9	0.71779 (18)	0.04083 (18)	0.9488 (3)	0.0490 (7)	
C14	0.77740 (19)	−0.02806 (19)	0.9807 (3)	0.0566 (8)	
H14A	0.7755	−0.0593	1.0695	0.068*	
C12	0.83890 (19)	0.00022 (19)	0.7535 (4)	0.0561 (8)	
H12A	0.8803	−0.0134	0.6876	0.067*	
C1	0.52589 (19)	0.1264 (2)	1.1329 (3)	0.0520 (8)	
C8	0.6525 (2)	0.05759 (19)	1.0535 (3)	0.0534 (8)	
H8A	0.6547	0.0243	1.1407	0.064*	
C13	0.83925 (19)	−0.05108 (18)	0.8837 (4)	0.0545 (8)	
C2	0.48863 (19)	0.20659 (19)	1.1417 (3)	0.0563 (8)	
H2B	0.5078	0.2513	1.0850	0.068*	
C3	0.4222 (2)	0.2205 (2)	1.2357 (4)	0.0610 (9)	
C4	0.3933 (2)	0.1543 (3)	1.3211 (4)	0.0703 (10)	
H4A	0.3489	0.1636	1.3834	0.084*	
C6	0.49656 (19)	0.0600 (2)	1.2181 (4)	0.0608 (9)	
H6A	0.5208	0.0057	1.2118	0.073*	
C7	0.3820 (2)	0.3038 (3)	1.2386 (4)	0.0792 (11)	
C5	0.4310 (2)	0.0748 (2)	1.3128 (4)	0.0710 (10)	
H5A	0.4126	0.0304	1.3710	0.085*	
C15	0.9008 (2)	−0.1292 (2)	0.9173 (4)	0.0705 (10)	
C17	0.8421 (3)	−0.2099 (2)	0.9016 (6)	0.1168 (16)	
H17A	0.8121	−0.2145	0.7989	0.175*	
H17B	0.7977	−0.2068	0.9704	0.175*	
H17C	0.8798	−0.2590	0.9257	0.175*	
C16	0.9728 (3)	−0.1349 (3)	0.8100 (7)	0.145 (2)	
H16A	0.9437	−0.1377	0.7065	0.217*	
H16B	1.0088	−0.1851	0.8332	0.217*	
H16C	1.0110	−0.0854	0.8229	0.217*	
N1	0.3495 (3)	0.3705 (2)	1.2362 (5)	0.1135 (13)	
C19	0.7872 (2)	0.1226 (2)	0.5690 (4)	0.0619 (9)	
C20	0.6964 (2)	0.1167 (2)	0.4636 (4)	0.0807 (11)	
H20A	0.6839	0.0581	0.4368	0.121*	
H20B	0.6998	0.1493	0.3728	0.121*	
H20C	0.6488	0.1388	0.5153	0.121*	
C21	0.8071 (2)	0.2170 (2)	0.6111 (4)	0.0871 (12)	
H21A	0.7609	0.2386	0.6665	0.131*	
H21B	0.8079	0.2497	0.5198	0.131*	
H21C	0.8650	0.2212	0.6733	0.131*	
C18	0.9497 (3)	−0.1256 (2)	1.0812 (5)	0.1196 (17)	
H18A	0.9058	−0.1221	1.1508	0.179*	
H18B	0.9882	−0.0763	1.0931	0.179*	
H18C	0.9857	−0.1761	1.1021	0.179*	
C22	0.8623 (3)	0.0909 (3)	0.4806 (5)	0.1176 (17)	
H22A	0.8515	0.0322	0.4529	0.176*	
H22B	0.9199	0.0960	0.5434	0.176*	
H22C	0.8625	0.1245	0.3901	0.176*	
H1A	0.618 (3)	0.155 (3)	0.865 (6)	0.166 (19)*	

### Atomic displacement parameters (Å^2^)

**Table:**

	*U*^11^	*U*^22^	*U*^33^	*U*^12^	*U*^13^	*U*^23^
O1	0.0747 (15)	0.0676 (14)	0.0544 (15)	0.0222 (12)	0.0120 (12)	0.0117 (12)
C10	0.0533 (18)	0.0542 (18)	0.0421 (19)	0.0053 (16)	0.0001 (15)	−0.0018 (15)
N2	0.0599 (16)	0.0620 (16)	0.0468 (17)	0.0058 (14)	0.0115 (13)	−0.0027 (13)
C11	0.0463 (17)	0.0592 (19)	0.0423 (19)	−0.0004 (16)	0.0064 (14)	−0.0020 (15)
C9	0.0556 (18)	0.0545 (18)	0.0364 (18)	0.0040 (16)	0.0047 (14)	−0.0008 (15)
C14	0.064 (2)	0.0587 (19)	0.0467 (19)	−0.0012 (17)	0.0041 (16)	0.0110 (16)
C12	0.0492 (18)	0.065 (2)	0.056 (2)	−0.0056 (16)	0.0124 (15)	−0.0055 (17)
C1	0.0488 (17)	0.064 (2)	0.0428 (19)	−0.0021 (17)	0.0063 (15)	−0.0084 (16)
C8	0.062 (2)	0.0577 (19)	0.0395 (18)	−0.0017 (17)	0.0056 (15)	−0.0024 (15)
C13	0.0469 (17)	0.0579 (19)	0.058 (2)	0.0023 (16)	0.0054 (15)	−0.0025 (17)
C2	0.058 (2)	0.060 (2)	0.052 (2)	−0.0047 (17)	0.0137 (16)	−0.0084 (16)
C3	0.058 (2)	0.068 (2)	0.058 (2)	−0.0018 (18)	0.0112 (17)	−0.0182 (19)
C4	0.055 (2)	0.095 (3)	0.065 (2)	−0.011 (2)	0.0217 (18)	−0.013 (2)
C6	0.057 (2)	0.063 (2)	0.062 (2)	−0.0070 (17)	0.0070 (17)	−0.0004 (18)
C7	0.081 (3)	0.077 (3)	0.085 (3)	−0.002 (2)	0.028 (2)	−0.023 (2)
C5	0.064 (2)	0.083 (3)	0.069 (2)	−0.013 (2)	0.0186 (19)	0.003 (2)
C15	0.059 (2)	0.065 (2)	0.085 (3)	0.0121 (18)	0.0026 (19)	0.0019 (19)
C17	0.108 (3)	0.067 (3)	0.166 (5)	0.005 (2)	−0.015 (3)	−0.008 (3)
C16	0.131 (4)	0.136 (4)	0.184 (5)	0.076 (3)	0.080 (4)	0.044 (4)
N1	0.120 (3)	0.088 (2)	0.140 (4)	0.008 (2)	0.046 (3)	−0.034 (2)
C19	0.061 (2)	0.075 (2)	0.051 (2)	−0.0047 (18)	0.0139 (17)	0.0107 (18)
C20	0.085 (2)	0.105 (3)	0.049 (2)	−0.011 (2)	−0.0005 (19)	0.007 (2)
C21	0.093 (3)	0.095 (3)	0.070 (3)	−0.032 (2)	−0.001 (2)	0.023 (2)
C18	0.105 (3)	0.099 (3)	0.138 (4)	0.030 (3)	−0.043 (3)	0.012 (3)
C22	0.115 (3)	0.160 (4)	0.090 (3)	0.033 (3)	0.061 (3)	0.045 (3)

### Geometric parameters (Å, °)

**Table:**

O1—C10	1.369 (3)	C7—N1	1.152 (4)
O1—H1A	1.03 (5)	C5—H5A	0.9300
C10—C9	1.404 (4)	C15—C17	1.535 (4)
C10—C11	1.405 (4)	C15—C16	1.530 (5)
N2—C8	1.286 (3)	C15—C18	1.533 (5)
N2—C1	1.431 (3)	C17—H17A	0.9600
C11—C12	1.395 (4)	C17—H17B	0.9600
C11—C19	1.543 (4)	C17—H17C	0.9600
C9—C14	1.403 (4)	C16—H16A	0.9600
C9—C8	1.456 (4)	C16—H16B	0.9600
C14—C13	1.391 (4)	C16—H16C	0.9600
C14—H14A	0.9300	C19—C22	1.534 (4)
C12—C13	1.406 (4)	C19—C20	1.537 (4)
C12—H12A	0.9300	C19—C21	1.545 (4)
C1—C2	1.382 (4)	C20—H20A	0.9600
C1—C6	1.391 (4)	C20—H20B	0.9600
C8—H8A	0.9300	C20—H20C	0.9600
C13—C15	1.534 (4)	C21—H21A	0.9600
C2—C3	1.396 (4)	C21—H21B	0.9600
C2—H2B	0.9300	C21—H21C	0.9600
C3—C4	1.387 (4)	C18—H18A	0.9600
C3—C7	1.438 (5)	C18—H18B	0.9600
C4—C5	1.374 (4)	C18—H18C	0.9600
C4—H4A	0.9300	C22—H22A	0.9600
C6—C5	1.390 (4)	C22—H22B	0.9600
C6—H6A	0.9300	C22—H22C	0.9600
			
C10—O1—H1A	108 (3)	C17—C15—C13	108.9 (3)
O1—C10—C9	119.5 (3)	C16—C15—C13	112.2 (3)
O1—C10—C11	119.6 (3)	C18—C15—C13	110.4 (3)
C9—C10—C11	120.9 (3)	C15—C17—H17A	109.5
C8—N2—C1	120.6 (3)	C15—C17—H17B	109.5
C12—C11—C10	116.1 (3)	H17A—C17—H17B	109.5
C12—C11—C19	121.9 (3)	C15—C17—H17C	109.5
C10—C11—C19	122.0 (3)	H17A—C17—H17C	109.5
C10—C9—C14	119.8 (3)	H17B—C17—H17C	109.5
C10—C9—C8	122.1 (3)	C15—C16—H16A	109.5
C14—C9—C8	118.1 (3)	C15—C16—H16B	109.5
C13—C14—C9	122.0 (3)	H16A—C16—H16B	109.5
C13—C14—H14A	119.0	C15—C16—H16C	109.5
C9—C14—H14A	119.0	H16A—C16—H16C	109.5
C11—C12—C13	125.8 (3)	H16B—C16—H16C	109.5
C11—C12—H12A	117.1	C22—C19—C20	108.2 (3)
C13—C12—H12A	117.1	C22—C19—C21	107.7 (3)
C2—C1—C6	119.5 (3)	C20—C19—C21	109.2 (3)
C2—C1—N2	116.9 (3)	C22—C19—C11	112.0 (3)
C6—C1—N2	123.6 (3)	C20—C19—C11	109.5 (2)
N2—C8—C9	123.1 (3)	C21—C19—C11	110.1 (3)
N2—C8—H8A	118.4	C19—C20—H20A	109.5
C9—C8—H8A	118.4	C19—C20—H20B	109.5
C14—C13—C12	115.5 (3)	H20A—C20—H20B	109.5
C14—C13—C15	121.1 (3)	C19—C20—H20C	109.5
C12—C13—C15	123.4 (3)	H20A—C20—H20C	109.5
C1—C2—C3	119.9 (3)	H20B—C20—H20C	109.5
C1—C2—H2B	120.0	C19—C21—H21A	109.5
C3—C2—H2B	120.0	C19—C21—H21B	109.5
C4—C3—C2	120.4 (3)	H21A—C21—H21B	109.5
C4—C3—C7	120.7 (3)	C19—C21—H21C	109.5
C2—C3—C7	118.9 (3)	H21A—C21—H21C	109.5
C5—C4—C3	119.4 (3)	H21B—C21—H21C	109.5
C5—C4—H4A	120.3	C15—C18—H18A	109.5
C3—C4—H4A	120.3	C15—C18—H18B	109.5
C5—C6—C1	120.1 (3)	H18A—C18—H18B	109.5
C5—C6—H6A	119.9	C15—C18—H18C	109.5
C1—C6—H6A	119.9	H18A—C18—H18C	109.5
N1—C7—C3	178.0 (4)	H18B—C18—H18C	109.5
C4—C5—C6	120.6 (3)	C19—C22—H22A	109.5
C4—C5—H5A	119.7	C19—C22—H22B	109.5
C6—C5—H5A	119.7	H22A—C22—H22B	109.5
C17—C15—C16	109.5 (3)	C19—C22—H22C	109.5
C17—C15—C18	107.9 (3)	H22A—C22—H22C	109.5
C16—C15—C18	107.9 (3)	H22B—C22—H22C	109.5

### Hydrogen-bond geometry (Å, °)

**Table:**

*D*—H···*A*	*D*—H	H···*A*	*D*···*A*	*D*—H···*A*
O1—H1A···N2	1.03 (5)	1.68 (5)	2.612 (3)	149 (4)
